# *P*. *aeruginosa* augments irradiation injury via 15-lipoxygenase–catalyzed generation of 15-HpETE-PE and induction of theft-ferroptosis

**DOI:** 10.1172/jci.insight.156013

**Published:** 2022-02-22

**Authors:** Haider H. Dar, Michael W. Epperly, Vladimir A. Tyurin, Andrew A. Amoscato, Tamil S. Anthonymuthu, Austin B. Souryavong, Alexander A. Kapralov, Galina V. Shurin, Svetlana N. Samovich, Claudette M. St. Croix, Simon C. Watkins, Sally E. Wenzel, Rama K. Mallampalli, Joel S. Greenberger, Hülya Bayır, Valerian E. Kagan, Yulia Y. Tyurina

**Affiliations:** 1 Department of Environmental and Occupational Health and Center for Free Radical and Antioxidant Health, University of Pittsburgh, Pittsburgh, Pennsylvania, USA.; 2 Department of Radiation Oncology, UPMC Hillman Cancer Center, Pittsburgh, Pennsylvania, USA.; 3 Department of Critical Care Medicine, Safar Center for Resuscitation Research, University of Pittsburgh Medical Center, Pittsburgh, Pennsylvania, USA.; 4 Department of Cell Biology, University of Pittsburgh, Pittsburgh, Pennsylvania, USA.; 5 Department of Internal Medicine, The Ohio State University, Columbus, Ohio, USA.; 6 Children’s Neuroscience Institute, Children’s Hospital of Pittsburgh, University of Pittsburgh, Pittsburgh, Pennsylvania, USA.; 7 Institute for Regenerative Medicine, I.M. Sechenov First Moscow State Medical University, Moscow, Russia.; 8 Departments of Pharmacology and Chemical Biology, Chemistry, Radiation Oncology, University of Pittsburgh, Pennsylvania, USA.

**Keywords:** Infectious disease, Inflammation, Bacterial infections, Chemokines, Radiation therapy

## Abstract

Total body irradiation (TBI) targets sensitive bone marrow hematopoietic cells and gut epithelial cells, causing their death and inducing a state of immunodeficiency combined with intestinal dysbiosis and nonproductive immune responses. We found enhanced *Pseudomonas aeruginosa* (PAO1) colonization of the gut leading to host cell death and strikingly decreased survival of irradiated mice. The PAO1-driven pathogenic mechanism includes theft-ferroptosis realized via (a) curbing of the host antiferroptotic system, GSH/GPx4, and (b) employing bacterial 15-lipoxygenase to generate proferroptotic signal — 15-hydroperoxy-arachidonoyl-PE (15-HpETE-PE) — in the intestines of irradiated and PAO1-infected mice. Global redox phospholipidomics of the ileum revealed that lysophospholipids and oxidized phospholipids, particularly oxidized phosphatidylethanolamine (PEox), represented the major factors that contributed to the pathogenic changes induced by total body irradiation and infection by PAO1. A lipoxygenase inhibitor, baicalein, significantly attenuated animal lethality, PAO1 colonization, intestinal epithelial cell death, and generation of ferroptotic PEox signals. Opportunistic PAO1 mechanisms included stimulation of the antiinflammatory lipoxin A_4_, production and suppression of the proinflammatory hepoxilin A_3_, and leukotriene B_4_. Unearthing complex PAO1 pathogenic/virulence mechanisms, including effects on the host anti/proinflammatory responses, lipid metabolism, and ferroptotic cell death, points toward potentially new therapeutic and radiomitigative targets.

## Graphical abstract

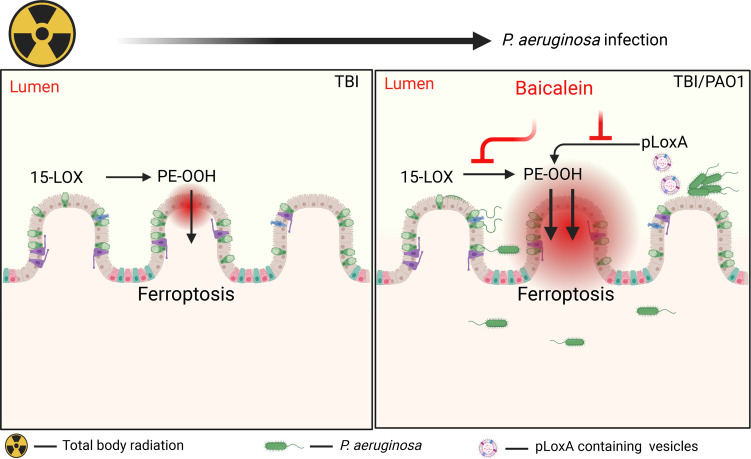

## Introduction

Exposure to ionizing radiation initiates generation of a multitude of reactive intermediates leading to DNA damage, and this triggers predominantly apoptotic death of sensitive cell populations — gut epithelium and bone marrow hematopoietic cells (1, 2). These events transition into a cascade of aberrant metabolic and immune responses, including recruitment/activation of innate immune cells, which create a prooxidant environment that facilitates the initiation of several types of nonapoptotic regulated cell death programs. These processes define the stage of sterile inflammatory responses to irradiation (3). The 2 perilous consequences of this — immunosuppression and breach of the intestinal epithelial barrier — pave the way for infection by bacterial pathogens leading to sepsis, multiple organ failure, and death (4).

The interaction of the gut microbiome and radiation is bidirectional whereby radiation can disrupt the microbiome by promoting dysbiosis, which in turn affects the body’s responses to irradiation (5). In this chain of events, intestinal dysbiosis favoring colonization by pathogenic bacterial strains, and their blossoming in immunocompromised conditions, plays a very important role (6). Among such strains, a Gram-negative bacterium, *Pseudomonas aeruginosa* (PAO1), is well-known to be particularly virulent and successful in immunosuppressed proinflammatory environments (7–9).

PAO1 possesses a unique toolkit of genes, the products of which hijack the host’s lipid metabolic pathways for the benefit of the pathogen (7). Our previous work has identified the PAO1 15-lipoxygenase (pLoxA) as a powerful tool used by the pathogen to selectively oxidize the host’s phospholipid, phosphatidylethanolamine (PE), and specifically the molecular species containing polyunsaturated fatty acids residues-PE (PUFA-PE), generating death signals in one of the recently discovered programs of cell death, ferroptosis (10–12). We further established that pLoxA-enriched vesicles secreted by PAO1 trigger ferroptosis in epithelial cells (12). This has been documented for the laboratory strain of the pathogen as well as for clinical isolates of PAO1 from immunocompromised patients (12). Notably, PAO1 intestinal carriage increases from approximately 3%–10% in healthy people to approximately 20%–30% in hospitalized immunocompromised patients (13–15). Based on these facts, we hypothesized that total body irradiation (TBI) induced immunosuppression and injury of the gut epithelium, triggering a vicious cycle in which intestinal dysbiosis selects highly virulent strains of PAO1 that utilize their pLoxA-driven mechanisms to induce ferroptosis, eliminate the intestinal epithelial barrier, and transition to PAO1 induced nonsterile inflammation.

In the current work, we discovered that PAO1 dramatically boosts the injury caused by TBI as evidenced by (a) markedly accelerated mortality of exposed mice, (b) enhanced recruitment and activation of innate immune cells, (c) production and release of proinflammatory cytokines and chemokines, (d) elevated levels of proinflammatory lipid mediators and oxidized phospholipids, (e) accumulation of myeloperoxidase-specific lipid metabolites, (f) activation of ferroptotic cell death, and (g) protective effects of a 15-lipoxygenase (15-LOX) inhibitor, baicalein. These aggressive features of PAO1 virulence position this strain as a dominant gateway species, facilitating penetration and spreading infection to cause sepsis and multiple organ failure after TBI.

## Results

### Infection with PAO1 markedly shortens the survival of irradiated mice.

**Figure 1:**
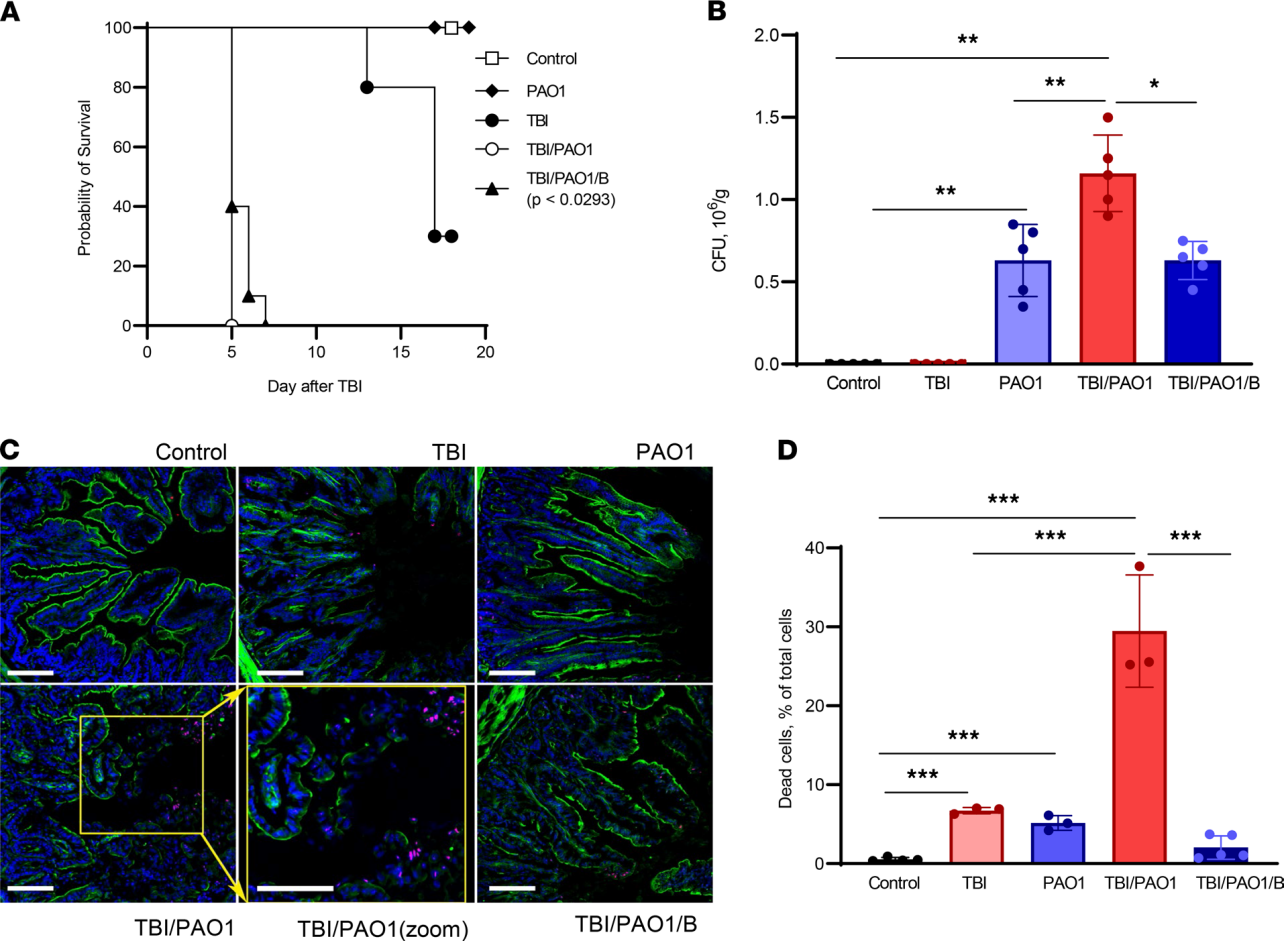
PAO1 alters the survival of irradiated mice. (**A**) C57BL/6J mice after TBI (9.25 Gy) were infected with PAO1 in presence or absence of lipoxygenase inhibitor (baicalein) and monitored for more than 15 days. Unirradiated (C) and unirradiated PAO1-infected (PAO1) mice served as controls. Data are pooled from 2 independent experiments. (**B**) PAO1 burden in colon. Fecal samples were collected, resuspended, and serially plated on cetrimide agar and incubated at 37°C for 20 hours. PAO1 colonies were counted and represented as CFU/g. Data represent mean ± SD, *n* = 5 mice/group. **P < 0.01; *P < 0.05, 1-way ANOVA, Tukey’s multiple comparison test. (**C**) Baicalein treatment mitigated epithelial barrier disruption induced by TBI plus PAO1. Damage was assessed as discontinuity of the actin layer (green), and cell death (pink nuclei) that was particularly evident at the apex of the crypt. Dead cell (red), F-actin (green), and Hoechst (blue). Scale: 50 μm. (**D**) Baicalein treatment mitigated cell death induced by TBI plus PAO1. TBI, total body irradiation; PAO1, *P*. *aeruginosa*; B, baicalein. Data represent mean ± SD, *n* = 5 mice/group for control, TBI/PAO1/B; *n* = 3, for TBI, PAO1, and TBI/PAO1. ***P < 0.001, 1-way ANOVA, Tukey’s multiple comparison test.

TBI of C57BL/6J mice to a dose of 9.25 Gy caused a typical 70% lethality on day 15. After administration of PAO1 the day after TBI, we observed an astonishing decrease in the survival of mice, such that 100% lethality occurred on day 5 after irradiation (Figure 1A). Infection of mice with PAO1 by itself did not cause any death within 18 days, similar to the control group of mice (Figure 1A). Further, we confirmed the presence of PAO1 in the fecal samples of mice collected on day 4 (1 day before the mice died) (Figure 1B). The amount of PAO1 was approximately 2 times higher in the group of mice preirradiated the day before the infection. These results suggest that PAO1 successfully colonized the irradiated animals and specific PAO1-induced changes in irradiated mice led to their early death. In our experimental set-up, the response of TBI-only treated mice was similar to the previously reported (16, 17). By contrast, exposure of TBI mice to PAO1 caused a drastic change in the survival (from 15 to 4 days) (Figure 1A). Therefore, we further focused on understanding the mechanisms responsible for PAO1-induced changes in the irradiated mice.

Rapidly proliferating gut epithelium is known to be highly sensitive to irradiation (2). Indeed, on day 4 after TBI, confocal microscopy revealed substantial damage to the intestinal epithelium, detectable by the interruptions of the actin coating of the villa (Figure 1C). In line with this, the epithelial injury was markedly increased in the TBI group infected with PAO1 (Figure 1C) as evidenced by the greater cell death (assessed by the tunnel assay) detected in irradiated mice treated with PAO1 compared to irradiated-only mice (Figure 1D).

PAO1 is known to produce and employ several virulence factors to execute the death programs in host cells (18). Among these factors, pLoxA was shown to be important for pathogen survival inside the host, as well as for inducing host cell death (18, 19). To explore a possible role of pLoxA in the PAO1-dependent decreased survival of irradiated mice, we performed the experiments in the presence of baicalein, a known potent inhibitor of mammalian lipoxygenases (20). We used LC-MS and assessed the suppressive effects of baicalein on pLoxA oxidation of free arachidonic acid (AA) as well as 1-stearoyl-2-arachidonoyl-phosphatidylethanolamine (1-SA-2-AA-PE) in a model system (Supplemental Figure 1, A–C; supplemental material available online with this article; https://doi.org/10.1172/jci.insight.156013DS1). We found that baicalein inhibited pLoxA at nanomolar concentrations, whereby its effectiveness against accumulation of proferroptotic HOO-AA-PE was about an order of magnitude higher than against free HOO-AA (Supplemental Figure 1, A–C). This is in line with our previous data showing an inhibitory effect of baicalein on pLoxA-stimulated ferroptosis in human bronchial epithelial cells in vitro (12, 20). Notably, we observed a significant albeit incomplete (40%) mitigation effect of baicalein on survival of irradiated mice on day 5 of treatment (Figure 1A). Importantly, baicalein markedly attenuated morphologically assessed injury of the intestinal epithelium (Figure 1C), decreased the numbers of dead cells (Figure 1D), and prevented irradiation-enhanced PAO1 colonization of the host gut (Figure 1B). These results suggest that the increased death of irradiated and PAO1-infected mice was due, at least in part, to the effects of pLoxA.

### Identification of characteristic/distinguishing phospholipid species by orthogonal projection to latent structures discriminant analysis.

**Figure 2:**
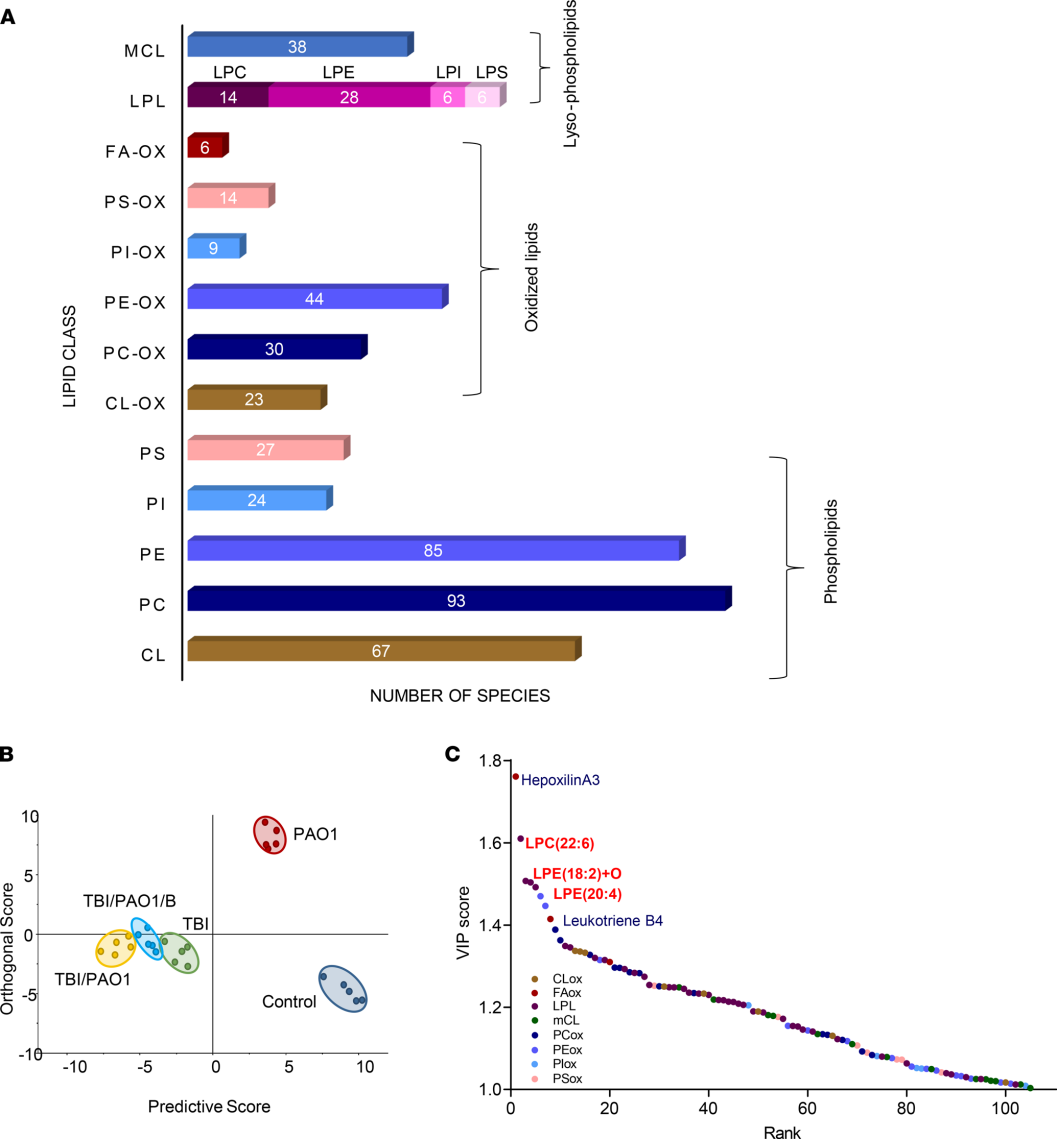
PAO1-induced changes in ileal lipidome of mice exposed to TBI. (**A**) Numbers of lipid species identified in mouse ileum. LPL, lysophospholipids; mCL, monolysocardiolipin; LPC, lysophospholipids; LPE, lysophosphatidylethanolamine; PS, phosphatidylserine; PI, phosphatidylinositol; PE, phosphatidylethanolamine; PC, phosphatidylcholine; CL, cardiolipin; FA-OX, oxidized fatty acids; PS-OX, oxidized phosphatidylserine; PI-OX, oxidized phosphatidylinositol; PE-OX, oxidized phosphatidylethanolamine; PC-OX, oxidized phosphatidylcholine; CL-OX, oxidized cardiolipin. (**B**) OPLS-DA score plots showing the lipidomic differences between various groups of mice. (**C**) Dot plot showing variable of importance (VIP) scores of lipids that substantially contributed to the segregation between different groups in OPLS-DA. TBI, total body irradiation; PAO1, *P*. *aeruginosa*; B, baicalein.

To gain deeper insight into the mechanism(s) of the strong response elicited by PAO1-infected irradiated mice, we performed global phospholipidomics and redox lipidomics analyses of the intestinal tissue on day 4 after TBI (Supplemental Figures 2–6 and Supplemental Table 1). We detected 787 different species of phospholipids — including 126 oxygenated species and 79 species of lysophospholipids (LPL) (Figure 2A). We paid particular attention to 4 groups of lipids related to inflammation: (a) oxidation products related to execution of cell death programs (particularly oxidized phosphatidylethanolamine (PEox) in ferroptosis) (10); (b) lysophospholipids generated via myeloperoxidase-dependent (MPO-dependent) hydrolysis of ether bonds in plasmalogens; (c) inflammation-associated oxidized phospholipids (e.g., oxidized phosphatidylcholine [PCox] and its truncated species); and (d) lipid mediators with pro- and antiinflammatory mechanisms of action (21, 22). Orthogonal projection to latent structures discriminant analysis (OPLS-DA) revealed that irradiated groups of mice (TBI, TBI/PAO1, and TBI/PAO1/B) were well-separated from nonirradiated mice (control and PAO1), suggesting that radiation exposure had a profound impact on the segregation pattern of different groups of mice (Figure 2B). Further, the analysis also suggested that PAO1 infection markedly modulated the lipidomic signature of irradiated mice, and these changes were diminished in the presence of baicalein (Figure 2B).

Next, we wanted to identify the individual phospholipid species or a class of phospholipids responsible for this segregation pattern of different groups of mice. We generated variable of importance (VIP) plots from the OPLS-DA with a threshold of 1 to identify the phospholipid species that considerably contributed to the segregation between the groups. We found that LPL (39%) was the main class of phospholipids that contributed to the distinct behavior, followed by oxidized species of phospholipids (PEox, 15%, and PCox, 14%) (Supplemental Figure 7). While examining the contribution of individual phospholipid species, we discovered that the top 5 metabolites responsible for the observed differences were 3 LPLs (docosahexaenoic acid–containing [DHA-containing] lysophosphatidylcholine [LPC-22:6], arachidonic acid–containing [20:4] lysophosphatidylethanolamines [LPE-20:4], and lysophosphatidylethanolamine-containing mono-oxygenated linoleic acid [LPE-18:2+O] and 2 lipid mediators (hepoxilin A3 [HxA_3_] and leukotriene B4 [LTB_4_]) (Figure 2C).

### Modulation of host immune lipid signaling by PAO1.

Regulation of the inflammatory responses through lipid-signaling is a multilayered process involving cross-talk between epithelial and immune cells (macrophages and neutrophils [PMN]) (23). Therefore, we examined the recruitment of PMN and macrophages in the small intestine. We observed an increase of PMN on day 2 after TBI versus nonirradiated controls (data not shown); this effect was more pronounced on day 4 after TBI (Supplemental Figure 8A). The contents of macrophages peaked on day 4 after TBI, and this effect was highest in the TBI/PAO1 group (Supplemental Figure 8B, left). Notably, M1 macrophages were prominent at this time point (Supplemental Figure 8B, right). These changes are compatible with a proinflammatory response in TBI and TBI/PAO1. Baicalein treatment reduced the recruitment of both PMN and macrophages in the TBI/PAO1 mice (Supplemental Figure 8, A and B).

**Figure 3:**
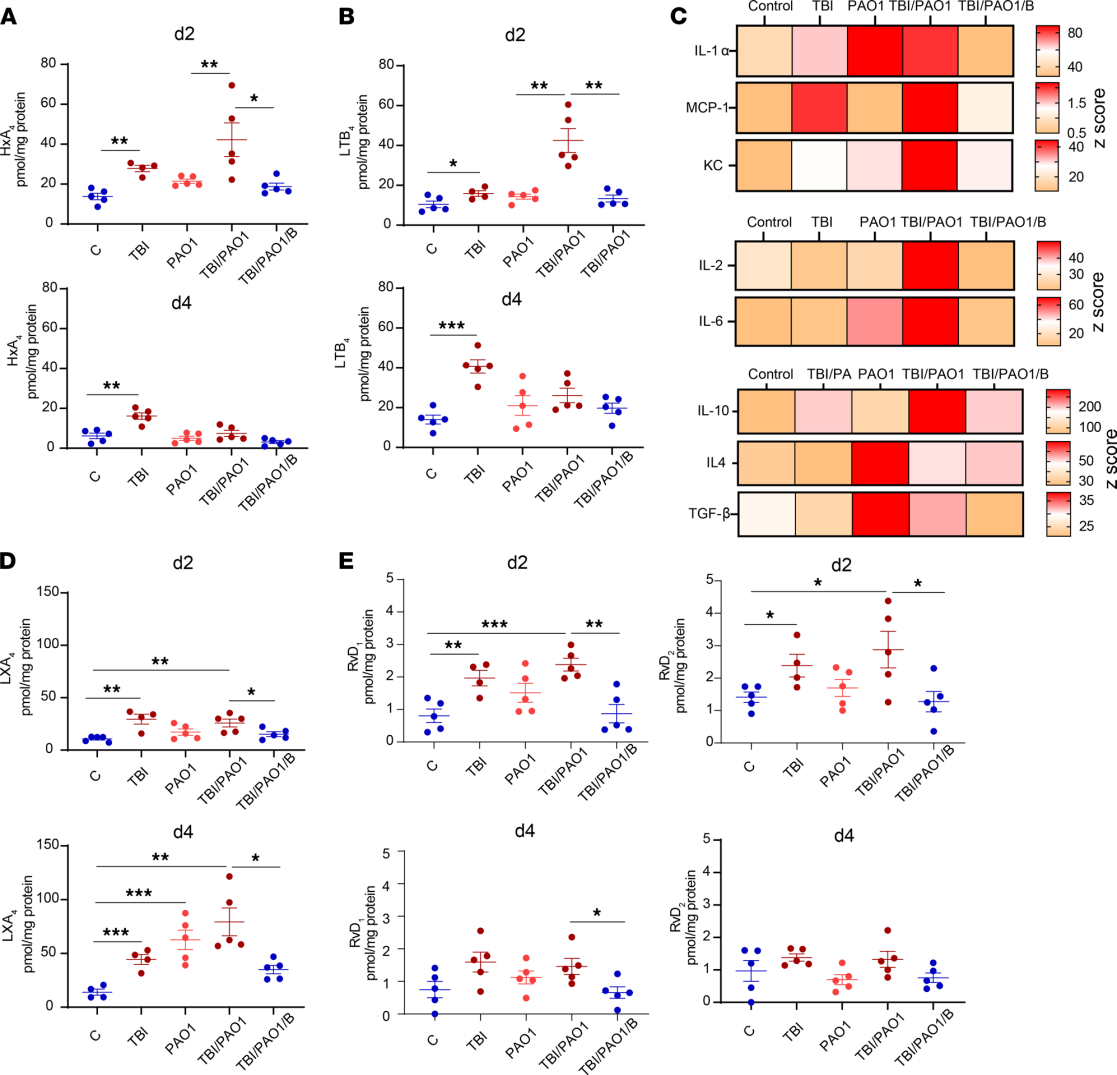
Administration of PAO1 modulates immune response of irradiated mice. (**A** and **B**) Quantitative assessment of proinflammatory lipid mediators HxA_3_ (**A**) and LTB_4_ (**B**) by LC-MS. (Ileal tissue homogenates from days 2 and 4 were processed and used for phospholipidomics analysis. Data represent mean ± SD, *n* = 5 mice/group. (**C**) Ileal tissue homogenates from day 2 were analyzed for cytokine and chemokine levels by Luminex assay; *n* = 5 mice/group. (**D** and **E**) LC-MS/MS-based quantification of proresolving lipid mediators LXA_4_ (**D**) and resolvins RvD_1_ and RvD_2_ (**E**). Data represent mean ± SD; *n* = 5 mice/group, d2 and d4 represent day 2 and day 4 after TBI. C, control; TBI, total body irradiation; PAO1, *P*. *aeruginosa*; B, baicalein; HxA_3_, hepoxilin A_3_; LTB_4_, leukotriene B_4_; LXA_4_, lipoxin A_4_; RvD_1_ and RvD_2_, resolvin D_1_ and D_2_, respectively. ***P < 0.001, **P < 0.01, *P < 0.05, 1-way ANOVA, Tukey’s multiple comparison test.

Evaluation of the levels of HxA_3_ and LTB_4_, the major proinflammatory lipid mediators regulating the recruitment and activation of PMNs, showed that in response to PAO1, the host enhanced the production of HxA_3_ and LTB_4_ (Figure 3, A and B, top). However, PAO1 strongly suppressed the generation of these proinflammatory lipid mediators on day 4 after TBI (Figure 3, A and B, bottom). Interestingly, the HxA_3_- and LTB_4_-aided proinflammatory milieu on day 2 after TBI was also supported by host cytokine response (Figure 3C). We found that the levels of proinflammatory chemoattractant cytokines like IL-1α, MCP-1, and KC, as well as cytokines produced by macrophages and PMN in response to infection (IL-1 and IL-6), were increased in TBI/PAO1-infected mice (Figure 3C). However, we also detected higher levels of a potent antiinflammatory cytokine, IL-10, in these mice, which in combination with TGF-β and IL-4 curbed the host immune response to the pathogen (Figure 3C). To further examine the interference of PAO1 with the host lipid signaling, we searched for proresolving lipid mediators. Significantly elevated levels of lipoxin A4 (LXA_4_), a potent antiinflammatory lipid mediator generated by the host 15-LOX, were found in the TBI and TBI/PAO1 groups versus control on day 2 after TBI (Figure 3D). Moreover, higher LXA_4_ levels were detected in the TBI/PAO1 group on day 4 after TBI (Figure 3D). In contrast, the change in LXA_4_ levels in TBI-treated groups on day 2 versus day 4 was minimal (Figure 3D). Further, in addition to AA, endogenous proresolving lipid mediators can also be generated from eicosapentaenoic acid (24) or DHA (25). We detected 2 resolvins, resolvin D_1_ (RvD_1_) and resolvin D_2_ (RvD_2_), which are produced from DHA (Figure 3E). Both resolvins were increased in TBI and TBI/PAO1 groups versus control on day 2 but not on day 4 (Figure 3E). Overall, these results point towards an interaction between proinflammatory and antiinflammatory lipid mediators generated in response to or by PAO1.

### PAO1 augments ferroptotic death in irradiated mice.

**Figure 4:**
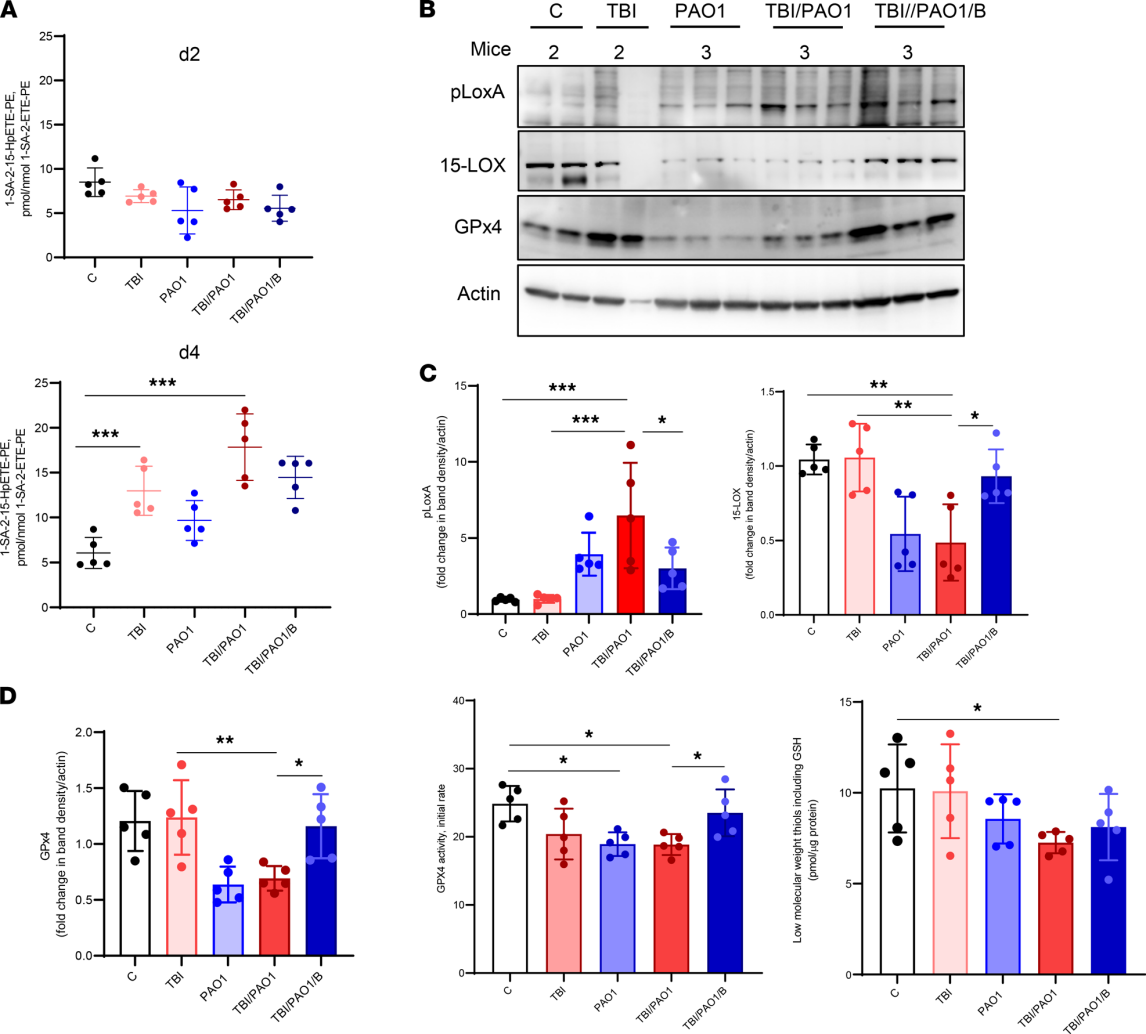
PAO1 enhances radiation-induced ferroptosis. (**A**) Levels of proferroptotic signal 1-stearoyl-2-15(S)-HpETE-sn-glycero-3-phosphoethanolamine (1-SA-2-15-HpETE-PE) were higher in irradiated mice after PAO1 infection. 24 hours after TBI, mice were left alone (TBI) or infected with PAO1 in the absence (TBI/PAO1) or presence of baicalein (TBI/PAO1/B). Ileum samples were collected on days 2 and 4 after TBI and processed for redox-lipidomics. Data represent mean ± SD, *n* = 5 mice/group; ***P < 0.0001, 1-way ANOVA, Tukey’s multiple comparison test. (**B**) Presence of 15-LOX, pLoxA, and GPx4 in ileum. Ileum homogenate from day 4 after radiation was tested for presence of 15-LOX, pLoxA, and GPx4 using antibody against 15-LOX and pLoxA. (**C**) Densitometry-based quantitative assessment of 15-LOX and pLoxA protein. Mean relative intensity of pLoxA (left) or 15-LOX (right) was normalized to actin. Data are mean ± SD, *n* = 5 mice/group; ***P < 0.0001; **P < 0.001; *P < 0.01, 1-way ANOVA, Tukey’s multiple comparison test. (**D**) Assessment of GPx4 protein and activity from day 4. Densitometry-based quantitative assessment of GPx4 (left). Mean relative intensity of GPx4 was normalized to actin. Data are mean ± SD, *n* = 5 mice/group; **P < 0.001; *P < 0.01, 1-way ANOVA, Tukey’s multiple comparison test. GPx4 activity measurements (middle). Ileal homogenates (15 μg of protein) after TBI were incubated in buffer containing 0.1 M Tris (pH = 8.0), 0.5 mM EDTA, 1.25% Triton X-100 with 1SA-2-15-HpETE-PE (30 μM), glutathione (150 μM), glutathione reductase (1 U/mL), and NADPH (35 μM). Activity was monitored by the disappearance of NADPH fluorescence (excitation wavelength: 340 nm; emission wavelength: 460 nm). Data are mean ± SD, *n* = 5 mice/group; *P < 0.05, 1-way ANOVA, Tukey’s multiple comparison test. Low-MW thiols and GSH content in gut homogenates on day 4. For calculation of GSH, fluorescence of Thiol probe IV disappearing after treatment with GPX were used. Data are mean ± SD, *n* = 5 mice/group; *P < 0.01 versus control, 1-way ANOVA, Tukey’s multiple comparison test.

Radiation exposure sets into motion multiple cell death programs including apoptosis, necroptosis, and ferroptosis (16, 17, 26). To assess the degree of ferroptotic cell death after TBI, we performed redox-phospholipidomics analyses of ileal tissue on days 2 and 4. We observed increased generation of a proferroptotic death signal, 1-stearoyl-2 hydroxyarachidonoyl-PE (1-SA-2-15-HpETE-PE), in only 2 groups of mice — TBI-only and TBI/PAO1 (Figure 4A and Supplemental Figure 9). No significant accumulation of the signal was detected on day 2, suggesting that development of ferroptosis is a relatively late event in TBI-induced injury (Figure 4A, top). We previously reported that PAO1 utilizes pLoxA stimulated ferroptosis as a part of its pathogenic strategy (12).

Therefore, to examine whether the enhanced generation of the ferroptotic signal in TBI/PAO1 mice is driven by pLoxA, we confirmed the presence of pLoxA by Western blot in the samples (Figure 4, B and C, left). Notably, assessment of the host 15-LOX in the samples revealed an approximately 2-fold decrease in the presence of PAO1 (Figure 4, B and C, right). Thus, pLoxA is predominantly involved in the generation of lipid-derived ferroptotic cell death signal, 1-SA-2-15-HpETE-PE, in the ileum of infected and irradiated mice. Further, we performed experiments in which intestinal epithelial cells (HIEC6 and Caco2) were coincubated with PAO1 strains expressing different levels of pLoxA: (a) wild type (PAO1-WT), (b) pLoxA overexpressing strain (PAO1-Δ*wsp*F) and (c) pLoxA-deficient strain (PAO1-*loxA:Tn*) (12, 27). We found that only pLoxA-containing strains of PAO1 (PAO1-WT and PAO1-Δ*wsp*F) induced ferroptosis, in sharp contrast to the pLoxA-deficient strain (PAO1-*loxA:Tn*) (Supplemental Figure 10) (12, 27, 28).

The selenoenzyme glutathione peroxidase 4 (GPx4) is the main endogenous antiferroptotic shield of the host that regulates ferroptosis by eliminating the accumulation of proferroptotic signals 1-SA-2-15-HpETE-PE (10). In our previous studies, we demonstrated that PAO1 triggers chaperone-mediated autophagy and elimination of GPx4 as a part of its proferroptotic strategy (28). Indeed, we found that ileal GPx4 expression and enzymatic activity were significantly lower in PAO1-treated mice versus control on day 4 (Figure 4, B and D). Notably, the antiferroptotic protection by baicalein included the preservation of the GPx4 protein and its enzymatic activity (Figure 4, B and D).

## Discussion

Gut microbiota are diverse communities of microorganisms, mostly anaerobic commensal nonpathogenic bacteria, which have coevolved and formed a multilayered mutually beneficial relationship with the host (29, 30). Among the various essential functions of the gut microbiome is the ability to prevent the colonization by exogenous bacteria as well as outgrowth or infection with Gram-negative pathogenic bacteria, through the phenomenon of colonization resistance (29, 30). Perturbance of this balance, known as dysbiosis, leads to loss of important functions and is a gateway for the emergence of hitherto undetectable opportunistic pathogenic bacteria (31).

One such factor responsible for the gut dysbiosis is exposure to ionizing γ radiation, both therapeutic and accidental (32). Radiation therapy, in addition to surgery and chemotherapy, remains a core treatment regimen for cancer and is used in 50% of patients, with a vital role in 25% of cancer cures (33). However, because of rapid cellular turnover, gut epithelium is highly sensitive to injury induced by anticancer radiation therapy against tumors of the pelvis, abdomen, or colorectal system (33). This local radiation-incited epithelial damage, along with dysbiosis and propagated by a proinflammatory environment, leads to an immunocompromised state of the gut (32). After TBI, the injury and death of hematopoietic cells strongly enhances the immunodeficiency (2, 34). In this study, we attempted to investigate the impact of an opportunistic bacterial pathogen on irradiated gut using a double hit mouse model of TBI and PAO1 infection. As reported previously (14), infection with PAO1 alone did not affect the survival of mice, suggesting the protective effect of the gut microbiome (Figure 1). However, when this balance was disturbed by irradiation, infection with PAO1 became detrimental for the mice and led to a drastic decrease of survival from 15 to 4 days (Figure 1).

**Figure 5:**
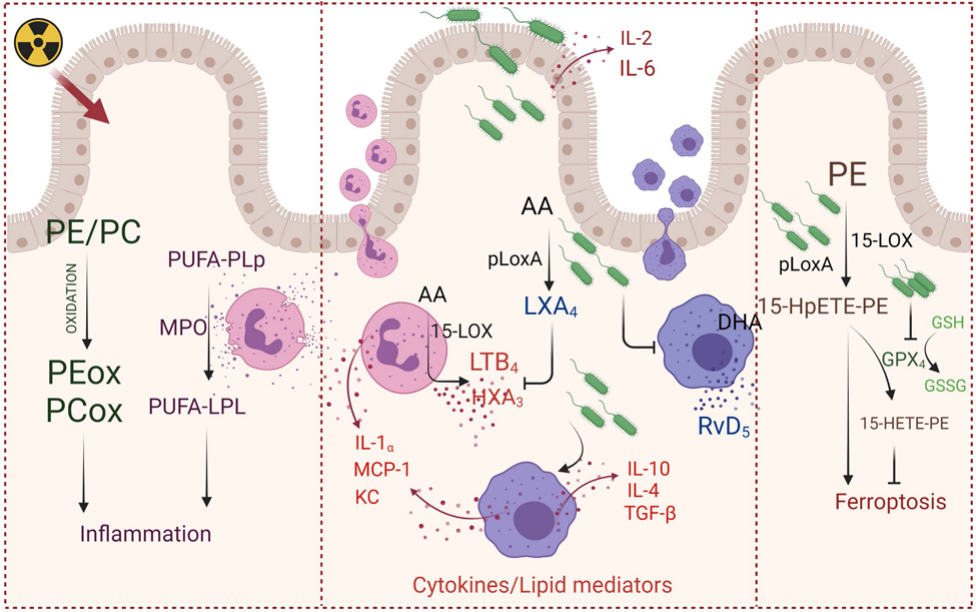
PAO1 boosts the injury of intestine epithelium caused by TBI. Left: TBI induces inflammatory response associated with accumulation of oxidized phospholipids and myeloperoxidase-specific lipid metabolites. Middle: PAO1 enhances TBI-induced recruitment and activation of innate immune cells (neutrophils and macrophages), accompanied by production and release of proinflammatory cytokines, chemokines, proinflammatory arachidonic acid–derived lipid mediators. Right: PAO1 augments generation of ferroptotic cell death signal, 15-HpETE-PE, utilizing pLoxA and suppressing GPx4 expression and enzymatic activity, hence activating ferroptotic cell death and markedly accelerating mortality of irradiated mice. PE, phosphatidylethanolamine; PC, phosphatidylcholine; PEox, oxidized phosphatidylethanolamine; PCox, oxidized phosphatidylcholine; PUFA-PLp, polyunsaturated acid containing plasmalogens; PUFA-LPL, lysophospholipids containing polyunsaturated fatty acids; 15-HpETE-PE, 15-hydroperoxyarachidonoyl-PE; 15-HETE-PE, 15-hydroxyarachidonoyl-PE; AA, arachidonic acid; LXA_4_- Lipoxin A4; LTB_4_, leukotriene B_4_; HXA_3_, hepoxilin A_3_; DHA, docosahexaenoic acid; RvD_5_, resolvin D_5_; MPO, myeloperoxidase; 15-LOX, host 15-lipoxygenase; pLoxA, a bacterial 15-lipoxygenase; GPX_4_, glutathione peroxidase 4; GSH, reduced glutathione; GSSG, oxidized glutathione. TBI, total body irradiation; PAO1, *P*. *aeruginosa*. Created with BioRender.com.

Although other opportunistic pathogens may also lead to infection in immunocompromised patients, we selected PAO1 in our study because it is one of the most commonly harbored pathogens in hospital settings, with the highest mortality rate among all opportunistic Gram-negative infections (8, 35). Further, the PAO1 strains of the gut are more virulent, their presence increases many-fold after irradiation, and they are the main source of sepsis-induced death even in the absence of any extraintestinal infection or bacteremia (8, 9, 36). PAO1 is unique as it has the ability to survive under the hyperinflammatory environment of the host (37). One pathway targeted and manipulated by pathogens is lipid mediator–stimulated host immune response. For example, *Mycobacterium tuberculosis* enhances synthesis of lipoxins (38) and inhibits a pro-inflammatory lipid mediator LTB_4_ (39). In this context, PAO1, with relatively higher content of regulatory genes and repertoire of secreted virulence factors including lipoxygenases, phospholipases, and epoxide hydrolase to target the host lipid metabolic pathways, may be distinctively posed to utilize both pro- and antiinflammatory approaches to thwart host responses (7, 40–42). Our work demonstrates that the host’s oxidized phospholipids play an important role in the responses of mice to different treatments (Figure 2). PAO1 modified the interactions between the host’s pro- and antiinflammatory signals — lipid mediators and cytokines — to gain the benefits of maximized colonization efficiency (Figures 1 and 3). Although we found that PAO1 enhanced the synthesis of an antiinflammatory LXA_4_, the complex interactions between several other antiinflammatory lipid mediators (e.g., resolvins) and a multitude of cytokines/chemokines defining the overall balance of the inflammatory regulators remains to be further explored (Figure 5).

In addition to manipulation of host lipid mediator–driven responses, recent studies have identified exploitation of host lipid peroxidation pathways as a virulence strategy of PAO1 (12, 43, 44). The process of ferroptosis is driven by peroxidation of PUFA-phospholipids and accumulation of unstable hydroperoxy-phospholipids — which can be cleaved to toxic levels of oxidatively truncated electrophilic products attacking nucleophilic sites in target proteins — if the regulatory reduced glutathione (GSH)/GPx4 system fails to reduce them to the more stable alcohols (11, 45). Execution of ferroptosis requires sufficient amounts of substrates, phospholipids with PUFA residues regulated by phospholipid remodeling enzymes like acyl-coenzyme A synthetase long chain family member 4 (ACSL4) and lysophosphosphatidylcholine acyltransferase 3 (LPCAT3) (10, 46, 47). In our previous studies, we demonstrated that PAO1-induced theft-ferroptosis executed by the secreted pLoxA (a) hijacks the host phospholipid peroxidation pathway whereby ACSL4 and LPCAT3 act as important regulators of the ferroptotic response and (b) targets the host GPx4 degradation by activating chaperone-mediated autophagy pathway (12, 28). Of note, the concept of theft-ferroptosis exploited as a pathogenic mechanism by *P*. *aeruginosa* has been supported by 2 recent independent studies, by Bagayoko et al. and Ousingsawat et al., which also highlighted and documented the critical role of *P*. *aeruginosa* induced ferroptosis in the pathology of pneumonia and cystic fibrosis (43, 44). Here, by using a lipoxygenase inhibitor, baicalein, we demonstrate that this increased lethality of PAO1 infection after irradiation is due, at least in part, to pLoxA-stimulated ferroptosis. This is further supported by our results showing depletion of the host GPx4 protein/activity and 15-LOX in the presence of pLoxA, which is prevented by baicalein (Figure 4, B and D). Baicalein also has antiinflammatory or antioxidant activities (48, 49). However, in conformity with an earlier report that identified baicalein as an antiferroptosis inhibitor by preventing GPx4 degradation and lipid peroxidation (50), our results emphasize the role of pLoxA-induced ferroptosis as a pathogenic factor in the enhanced lethality of TBI mice infected with PAO1.

In a recent elegant study, Guo et al. demonstrated the significance of gut microbiota against radiation-induced injury (51). This work established that the “elite-survivors” group of irradiated animals harbored a distinct gut microbiome compared with nonsurvivors and this distinct microbial community was solely responsible for the radioprotective effect (51). In sharp contrast, our study demonstrates an opposite microbiome-dependent effect: a severe worsening of the outcome of radiation injury. We demonstrate that the presence of an opportunistic pathogen, PAO1, in the gut synergistically enhances the lethality of irradiation. This damaging effect of PAO1 is realized, to a significant level, by its ability to blossom under proinflammatory conditions by using an antiinflammatory strategy in the production of lipid mediators and cytokines as well as expressing a proferroptotic 15-LOX, pLoxA, as a virulence factor (Figure 5). Our previous work and the current data identified a strong positive correlation between pLoxA activity of tobramycin-resistant *P*. *aeruginosa* isolates from patients with lower respiratory infection and ferroptosis of bronchial epithelial cells, thus emphasizing the importance of pLoXA-triggered proferroptotic lipid peroxidation in infectious diseases. This suggests that pLoxA inhibitors may have potential to significantly improve inflammation and prevent lipid peroxidation–driven pathogenesis and epithelial breach in cystic fibrosis, bacterial pneumonia, and immunocompromised cancer patients. In the context of radiation injury, our findings indicate that selective targeting of pLoxA may be a promising radiomitigative approach aimed at the reduction of the severe fatality of TBI combined with PAO1 infection.

## Methods

### Reagents.

Cetrimide agar (MilliporeSigma, 22470), lysogeny broth (LB) (MilliporeSigma, L3152), baicalein (MilliporeSigma, 465119), ferrostatin-1 (MilliporeSigma, SML0583), NADPH (MilliporeSigma), glutathione peroxidase (MilliporeSigma G6137), cumene hydroperoxide (MilliporeSigma C0524), Thiol Fluorescent Probe IV (MilliporeSigma 595504), Pierce BCA Protein Assay Kit (Thermo Fisher Scientific, 23225), anti-GPx4 (rabbit monoclonal, Abcam, ab125066), anti–15-LOX (rabbit polyclonal, Abcam, ab23691), anti-actin (mouse monoclonal, MilliporeSigma, A3854, clone AC-15), and secondary antibody, goat anti-rabbit (MilliporeSigma, A0545) were used. pLoxA antibody was purified from rabbit whole blood by Pocono Rabbit Farm and Laboratory as described previously (12, 27).

### Mice.

C57BL/6J female mice (7–8 weeks) were from Jackson Laboratories and were housed under specific pathogen–free conditions at the Animal Research Facility, University of Pittsburgh.

### Cells.

Human intestinal epithelial cell lines Caco2 (HTB-37) and HIEC6 (CRL-3266) purchased from ATCC were cultured in EMEM or MEM supplemented with 20% or 10% fetal calf serum (Gibco) and 100 U/mL penicillin-streptomycin (Thermo Fisher Scientific) incubated at 37°C and 5% CO_2_.

### Bacterial strains.

*P*. *aeruginosa* strains PAO1-WT, hyper-biofilm (Δ*wsp*F), and pLoxA-deficient (*loxA:Tn*) were obtained from a mutant library through the University of Washington, Seattle (12).

### TBI and PAO1 infection.

To evaluate the effect of PAO1 on TBI-induced intestinal damage, host microflora was reduced in all used mice by treatment with antibiotics in drinking water: ampicillin (1 g/L), vancomycin (0.5 g/L), neomycin trisulfate (1 g/L), and metronidazole (1 g/L) for 4 weeks. PAO1 colony was inoculated in 3 mL Luria-Bertani (LB) broth and incubated overnight (220 rpm) at 37°C. Then, 3 μL of this culture was re-inoculated in 3 mL of fresh LB broth and incubated for 18 hours. Then, the culture was centrifuged at 1200*g* for 15 minutes at 4°C and resuspended in 5 mL of PBS, diluted 1:100, and mice were infected with CFU of 1 × 10^7^ of PAO1. Mice were first irradiated (9.25 Gy), and then 24 hours after radiation exposure, an 18-gauge needle (Cadence Science, stainless steel, ball diameter of 0.023 cm and needle length of 2.54 cm) was used for gavage feeding of PAO1 directly into the stomachs of mice. One group of mice was injected i.p. with the 15-LOX inhibitor baicalein (50 mg/kg body weight in single-drug regimen) 24 hours after irradiation, before PAO1 gavage. Animals were sacrificed at days 2 and 4 after irradiation, and for survival studies, several groups of mice were maintained beyond 19 days. For those mice sacrificed at the early time points, intestine (ileum) was collected and analyzed for radiation-induced damage, levels of proinflammatory and stress response proteins (cytokines), pro- and antiinflammatory lipid mediators, and phospholipidomics.

### PAO1 burden in the gut.

Fecal samples were weighed and resuspended in peptone (1%) solution, diluted serially, and plated on a PAO1-specific cetrimide agar. Plates were incubated at 37°C overnight, and colonies were counted and expressed as CFU times 10^6^/g of fecal sample.

### Immunostaining and confocal microscopy.

Tissues (small intestine) were fixed in 2% paraformaldehyde at 4°C for 1–2 hours. The 5–7 μm sections were blocked with 5% donkey serum for 45 minutes and incubated for 40 minutes at 37°C with TUNEL (MilliporeSigma, TMR red 12156792910). Phalloidin (BioLegend 424201, 1:1000) was added for 30 minutes. Sections were labeled with Hoechst (MilliporeSigma B2883) 1 mg/100 mL dH_2_O and mounted using Gelvatol. High-resolution (20×/0.75NA) large-area montages (3 × 3 fields) were collected using a Nikon A1 confocal microscope equipped with GAsP detectors. Data acquisition and analysis were performed using NIS Elements software (Nikon Inc.).

### Isolation and dissociation of cells from small intestine and flow-cytometry analysis.

The small intestine of C57BL/6J mice was isolated, rinsed in cold PBS, opened longitudinally, and sliced into small fragments. First, epithelial cells and intraepithelial lymphocytes were disrupted from the mucosa by shaking the tissue at 250 rpm for 20 minutes at 37°C in HBSS containing 5% FBS and 2 mM EDTA. Cells were collected by filtering through 70 μm cell strainers. Then, the lamina propria tissue was further treated enzymatically with collagenase VIII (1.5 mg/mL)/DNAse I (40 μg/mL) in HBSS/5%FBS/2 mM EDTA solution at 200 rpm for 20 minutes at 37°C. Tissue debris was removed by filtering through 70 μm cell strainers. Both cell fractions were collected by centrifugation (500*g*, 10 minutes) followed by staining with a combination of fluorescence-labeled antibodies against the cell surface markers CD45 (BUV737, anti-mouse, clone 30-F11, BD Biosciences, 748371), CD11b (Brilliant Violet 605, anti-mouse, clone M1/70, BioLegend, 101257), F4/80 (pacific blue, anti-mouse, clone BM8, BioLegend, 123124), Ly6G (BV395, anti-mouse, clone 1A8, BD Biosciences, 563978), CD326 (PE/Cy7, anti-mouse, clone G8.8, BioLegend, 118216). Stained cells were washed twice in PBS containing 0.5% BSA and fixed with 1% PFA in PBS. Flow cytometric acquisition was performed on a Becton Dickinson LSR II instrument. Data were analyzed using FlowJo software (TreeStar, Inc.).

### Cytokine/chemokine quantification.

Cytokines/chemokines in intestinal tissue were measured by Luminex assay in triplicate according to the manufacturer’s instructions. To measure TGF-β1, a Single Plex Magnetic Bead Kit was utilized. To assess the levels of inflammatory mediator proteins in whole-tissue homogenates of ileum, a mouse 30-plex cytokine/chemokine microbead assay was used. The assays and kit were from MilliporeSigma.

### PAO1 supernatant collection.

Supernatant from different strains of PAO1 was collected as described previously (12). Briefly, after growing the strains in LB medium, at 37°C and 220 rpm for 14 hours, the strains were re-inoculated in MEM medium (without phenol red) at an OD600 of 0.05 in 96-well vinyl microtiter plates (100 μL per well, Costar). Plates were incubated at 37°C without agitation for 24 hours, and the supernatant was collected by centrifuging 2 times at 3,000 g for 8 minutes and then frozen before further use in cell culture experiments.

### Cell death.

Caco2 and HIEC6 cells were seeded (20,000/well) in 24-well plates. After 48 hours, cells were treated with supernatant from different strains of PAO1 (WT, Δ*wsp*F, and *loxA:Tn*) and ferrostatin 1 (0.4 μM). Cell death was estimated by LDH release assay after 20 hours of incubation as described previously (12).

### Redox lipidomics.

LC/ESI-MS analysis of lipids was performed on a Thermo HPLC system coupled to an Orbitrap Fusion Lumos mass spectrometer (Thermo Fisher Scientific). Phospholipids were separated on a normal phase column (Luna 3 μm Silica (2) 100 Å, 150 × 1.0 mm, Phenomenex) at a flow rate of 0.050 mL/minute. The column was maintained at 35°C. The analysis was performed using gradient solvents (A and B) containing 10 mM ammonium formate. Solvent A contained propanol/hexane/water (285:215:5, v/v/v), and solvent B contained propanol/hexane/water (285:215:40, v/v/v). The column was eluted for 0–3 minutes from 10%–37% solvent B, held at 37% solvent B for 3–15 minutes, followed by 37–100% solvent B from 15–23 minutes, held at 100% solvent B from 23 to 75 minutes, followed by a return to initial conditions over 76–90 minutes from 100 to 10% solvent B. The mass spectrometer was operated with an electrospray ionization probe in negative polarity mode and scanned from 400 to 1800 (*m/z*) at 120 K resolution in a data-dependent mode for MS/MS analysis. Ion source conditions were set as follows: spray voltage = 3.5 kV, sheath gas = 35 (AU), auxiliary gas = 17 (AU), sweep gas = 0 (AU), transfer tube temperature = 300°C, RF lens level = 65. Analysis of LC/MS data was performed using the Compound Discoverer software package (Thermo Fisher Scientific) with an in-house–generated analysis workflow and oxidized phospholipid database. Lipids were further filtered by retention time and confirmed by fragmentation MS. Deuterated phospholipids (Avanti Polar Lipids) were used as internal standards.

### Oxidation of AA and 1-SA-2-AA-PE by pLoxA in a model system.

Lipoxygenase activity of pLoxA was assessed by formation of primary products of AA oxidation — 15-hydroperoxy-arachidonic acid (15-HpETE) or 1-SA-2-15-HpETE-PE. Briefly, 400 nM pLoxA was incubated with unilamellar liposomes of 1,2-dioleoyl-phosphatidylcholine (DOPC)/1-SA-2-AA-PE (1:1) or DOPC/AA (1:1) (100 nm) in the presence of 0.5 μM H_2_O_2_ and 100 μM DTPA (for transition metal chelation) in 20 mM HEPES saturated with oxygen, pH 7.4, at 37°C, in the absence or in the presence different concentrations of baicalein. At the end of incubation, AA and PE as well as their oxygenated products were extracted by the Folch procedure and analyzed by LC-MS using reverse phase column C30 as previously described (52). LC/ESI-MS/MS analysis of lipids was performed on a Dionex HPLC system (utilizing the Chromeleon software), consisting of a Dionex UltiMate 3000 mobile phase pump, equipped with an UltiMate 3000 degassing unit and UltiMate 3000 autosampler (sampler chamber temperature was set at 4^o^C). The Dionex HPLC system was coupled to an Orbitrap Fusion Lumos mass spectrometer (Thermo Fisher Scientific).

### GPx4 activity.

Ileum homogenates were prepared in 20 mM HEPES buffer containing 100 μM DTPA and protease/phosphatase inhibitor (1X) using a Polytron PT1200E handheld homogenizer. After centrifugation of homogenates at 13,800*g* for 15 minutes, supernatants were collected and protein concentration was estimated using a BCA kit (Thermo Fisher Scientific, 23225). Reaction was performed using 15 μg of protein in buffer containing 0.1 M Tris (pH 8.0), 1.25% Triton X-100 with HpETE-PE (30 μM), GSH (150 μM), glutathione reductase (1 U/mL), and NADPH (35 μM). The amount of oxidized GSH was calculated based on the amount of NADPH consumed during GSSG reduction. Fluorescence of NADPH was measured at an excitation wavelength of 340 nm and emission wavelength of 460 nm using an RF-5301 spectrofluorometer (Shimadzu). Non-GPX4–dependent consumption of NADPH was assessed by decrease in fluorescence after incubation of samples without HpETE-PE and subtracted from each individual data.

### Low-MW thiols.

Low-MW thiols including GSH measurements were performed as described previously (12). Briefly, cells were resuspended in 50 μL of PBS lysed by freeze-thaw. Samples of 10 μL in triplicate were incubated with 10 μM Thiol Fluorescent Probe IV (MilliporeSigma) for 5 minutes and then analyzed with excitation and emission wavelengths of 400 nm and 465 nm using the Cytation 5 imaging reader (BioTek). Amounts of low-MW thiols were normalized by the protein concentration measured by the BCA method.

### Western blotting.

Ileum homogenates prepared using a Polytron PT1200E handheld homogenizer in buffer (20 mM HEPES, 100 μM DPTA) containing freshly added 1X protease/phosphatase inhibitor. The homogenate was diluted in 4X Laemmli buffer before loading in 8%–16% Tris-glycine gradient gels (Life Technologies). After transfer to a PVDF membrane, the membrane was blocked with PBST (5% milk) for 1 hour and then incubated with anti-pLoxA (1:1000), anti–15-LOX (1:1000), or anti-Gpx4 (1:1000) antibodies overnight and anti–β-actin (1:5000 for 1 hour) at room temperature. Membranes were washed with PBST 3 times, then incubated with HRP-conjugating goat anti-rabbit (1:1000) for 1 hour, and then developed with SuperSignal West Pico Chemiluminescent Substrate (Thermo Fisher Scientific) using Amersham Imager 600 (GE Health Care, Life Sciences). Quantification of the bands was performed using ImageJ software (NIH). Full uncut gels are available online as supplemental material.

### Statistics.

The data in figure legends are presented as mean ± SD values from at least 5 mice. In general, the exact value of the sample size (*n*) is presented in the figure legends. Statistical analyses were performed by 1-way ANOVA and Tukey’s multiple comparisons test, unless otherwise specified. *P* < 0.05 was considered to be statistically significant. Statistical analysis was performed with GraphPad Prism 9.2.0.

### Study approval.

All experimental procedures were conducted in accordance with the guidelines and policy set forth by the Institute of Laboratory Animal Resources, National Research Council, and approved by the protocols established by the Institutional Animal Care and Use Committee of the University of Pittsburgh (protocol 18022000).

## Author contributions

HHD, HB, VEK, and YYT conceived the study. HHD and ABS performed PAO1 burden experiments. MWE did radiation exposure and PA infection. VAT and AAA analyzed lipid mediators. TSA, SNS, and YYT performed lipidomics experiments and analyzed the data. TSA and YYT performed OPLS-DA analysis. GVS, AAK, and ABS isolated neutrophils and macrophages from intestine and analyzed the data. AAK did GSH measurements and analyzed the data. CMSC and SCW performed immunostaining and confocal imaging experiments and analyzed the data. SEW, RKM, and JSG participated in formulating the idea and interpreting the results. HB participated in writing the manuscript. HHD, YYT, and VEK wrote the manuscript. All authors read and approved the final manuscript.

## Supplementary Material

Supplemental data

## Version 1 01/18/2022

In-Press Preview

## Version 2 02/22/2022

Electronic publication
