# Supplementary material for: P. aeruginosa augments irradiation injury via 15-lipoxygenase–catalyzed generation of 15-HpETE-PE and induction of theft-ferroptosis
Source: JCI Insight. 2022 Feb 22;7(4):e156013. doi: 10.1172/jci.insight.156013 (PMC8876480; doi:10.1172/jci.insight.156013)

P. aeruginosa augments irradiation injury via 15-lipoxygenase catalyzed generation of 15-HpETE-PE and induction of theft-ferroptosis.

Haider H. Dar[1], Michael W. Epperly[2], Vladimir A. Tyurin[1], Andrew A. Amoscato[1], Tamil S. Anthonymuthu[1,3], Austin B. Souryavong[1], Alexander A. Kapralov[1], Galina V. Shurin[1], Svetlana N. Samovich[1], Claudette M. St. Croix[4], Simon C. Watkins[4], Sally E. Wenzel[1], Rama K. Mallampalli[5], Joel S. Greenberger[2], Hülya Bayır[1,3*], Valerian E. Kagan[1,6,7*], Yulia Y. Tyurina[1*]

Supplementary Information

Supplementary Table 1. Description of the abbreviations used in the Main text and Figures and Supplementary figures.

Abbreviation	Description	Abbreviation	Description
PE	phosphatidylethanolamine	AA	arachidonic acid
PC	phosphatidylcholine	DHA	docosahexaenoic acid
PS	phosphatidylserine	FA-ox	oxidized fatty acids
PI	phosphatidylinositol	PAO1	*Pseudomonas aeruginosa*
CL	cardiolipin	pLoxA	bacterial 15-lipoxygenase
PE-ox	oxidized phosphatidylethanolamine	15-LOX	15-lipoxygenase
PC-ox	oxidized phosphatidylcholine	1-SA-2-HpETE-PE	1-stearoyl-2-15(S)-HpETE-sn-glycero-3 phosphoethanolamine
PS-ox	oxidized phosphatidylserine	PUFA-PLp	Polyunsaturated fatty acid containing plasmalogen
PI-ox	oxidized phosphatidylinositol	LXA_4	Lipoxin A4
CL-ox	oxidized cardiolipin	LTB_4	leukotriene B_4
LPL	lyso-phospholipids	HXA_3	hepoxillin A_3
LPC	lyso-phosphatidylcholine	RvD_1	resolvin D_1
LPE	lyso-phosphatidylethanolamine	RvD2	resolvin D_2
LPS	lyso-phosphatidylserine	GPX_4	glutathione peroxidase 4
mCL	monolysocardiolipin	GSH	reduced glutathione
CN	carbon number	GSSG	oxidized glutathione
DB	double bond	B	baicalein
PUFA	polyunsaturated fatty acids	TBI	total body irradiation
PMN	neutrophils	ASCL4	acyl coenzyme A synthetase long chain family member 4
OPLS-DA	orthogonal projection of latent structures discriminant analysis	LPCAT3	lyso-phosphosphatidylcholine acyltransferase 3

A

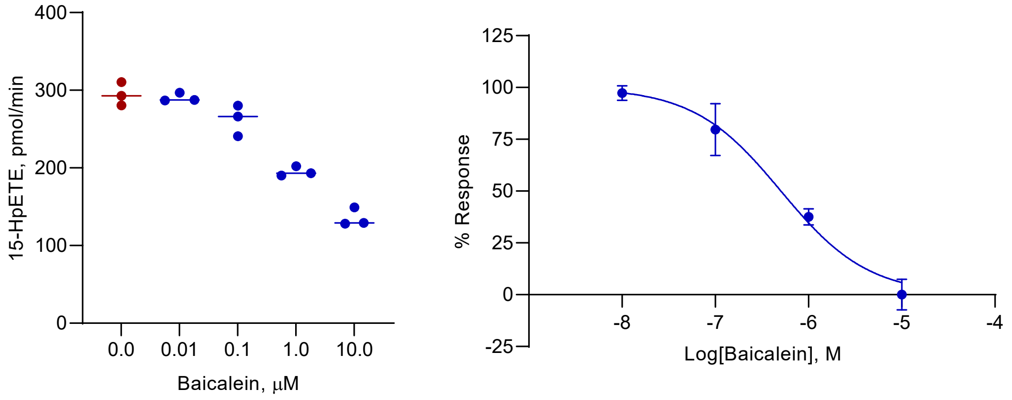

B

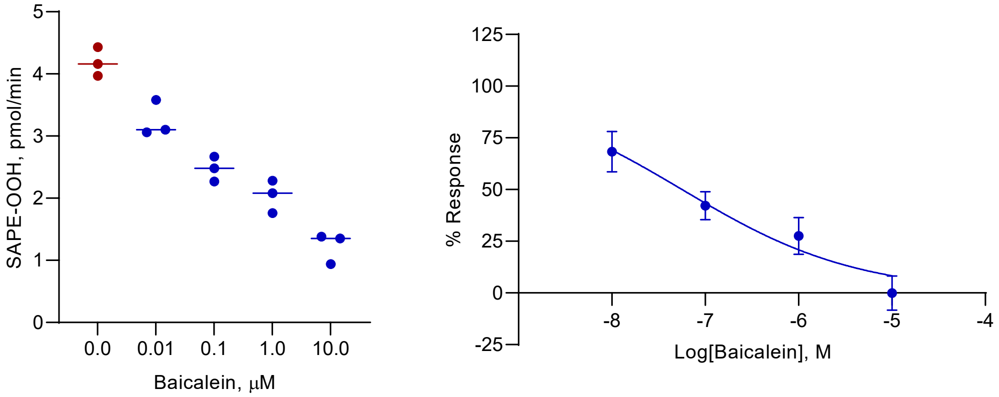

C

	**15-HpETE**	**1-sn-stearoyl-2-sn-15-HpETE-PE**
Initial rate, pmol/min	294.7 $\pm$ 15.1	4.2 $\pm$ 0.2
IC50 (95%CI) nM	506.4 (333.6-750.8)	56.5 (24.8-115.4)

Supplementary Figure 1. Inhibition of pLOXA activity by baicalein in model system. Related to Figure 1.(**A**). Initial rate of 15-HpETE generation induced by pLoxA in the absence and in the presence of baicalein (left panel). Inhibition of 15-HpETE accumulation catalyzed by pLoxA in the presence of baicalein (right panel). (**B**). Initial rate of stearoyl-sn-1-15-HpETE-sn-2-PE generation induced by pLoxA in the absence and in the presence of baicalein (left panel). Inhibition of stearoyl-sn-1-15-HpETE-sn-2-PE accumulation catalyzed by pLoxA in the presence of baicalein (right panel). (**C**) Initial rates for generation of 15-HpETE and stearoyl-sn-1-15-HpETE-sn-2-PE by pLoxA and calculated IC_{50} for baicalein required for 50% inhibition of pLoxA driven reactions.

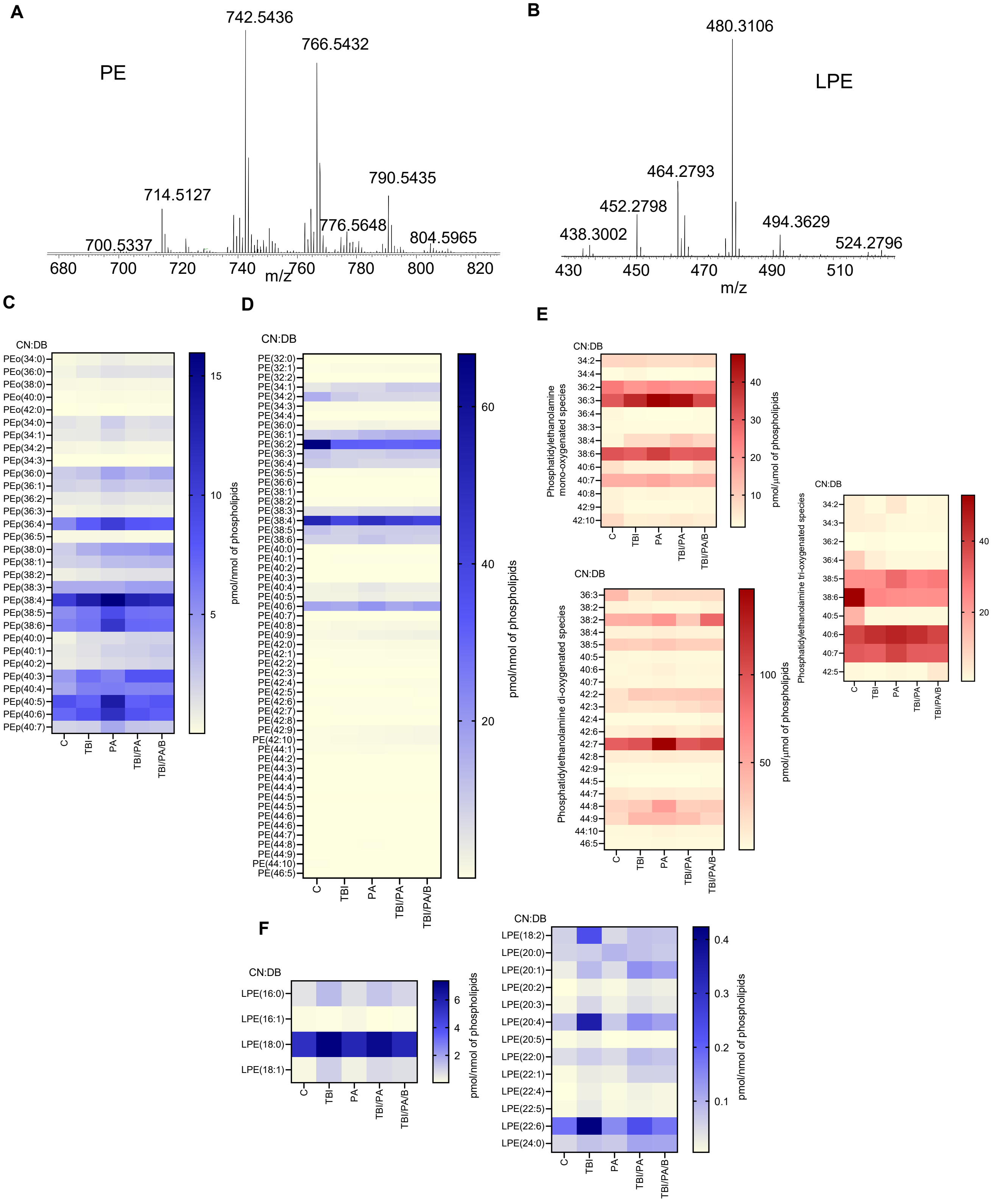

Supplementary Figure 2. Related to Figure 2. Typical full mass spectra of phosphatidylethanolamine (PE) (**A**) and lyso-phosphatidylethanolamine (LPE) (**B**) obtained from ileum of control mouse. Spectra were acquired in negative mode. Heat maps showing the content of PE plasmalogen (**C**), PE di-acyl (**D**), oxygenated PE (**E**) and LPE (lyso-PE) (**F**) molecular species in ileum of mice. n=5. PE, oxygenated PE and LPE species are labeled as CN:DB where CN is carbon number in sn-1 and sn-2 positions and DB is a double bond number in acyl chains. C -control; TBI - total body irradiation; PAO1 – *P. aeruginosa*; B – baicalein.

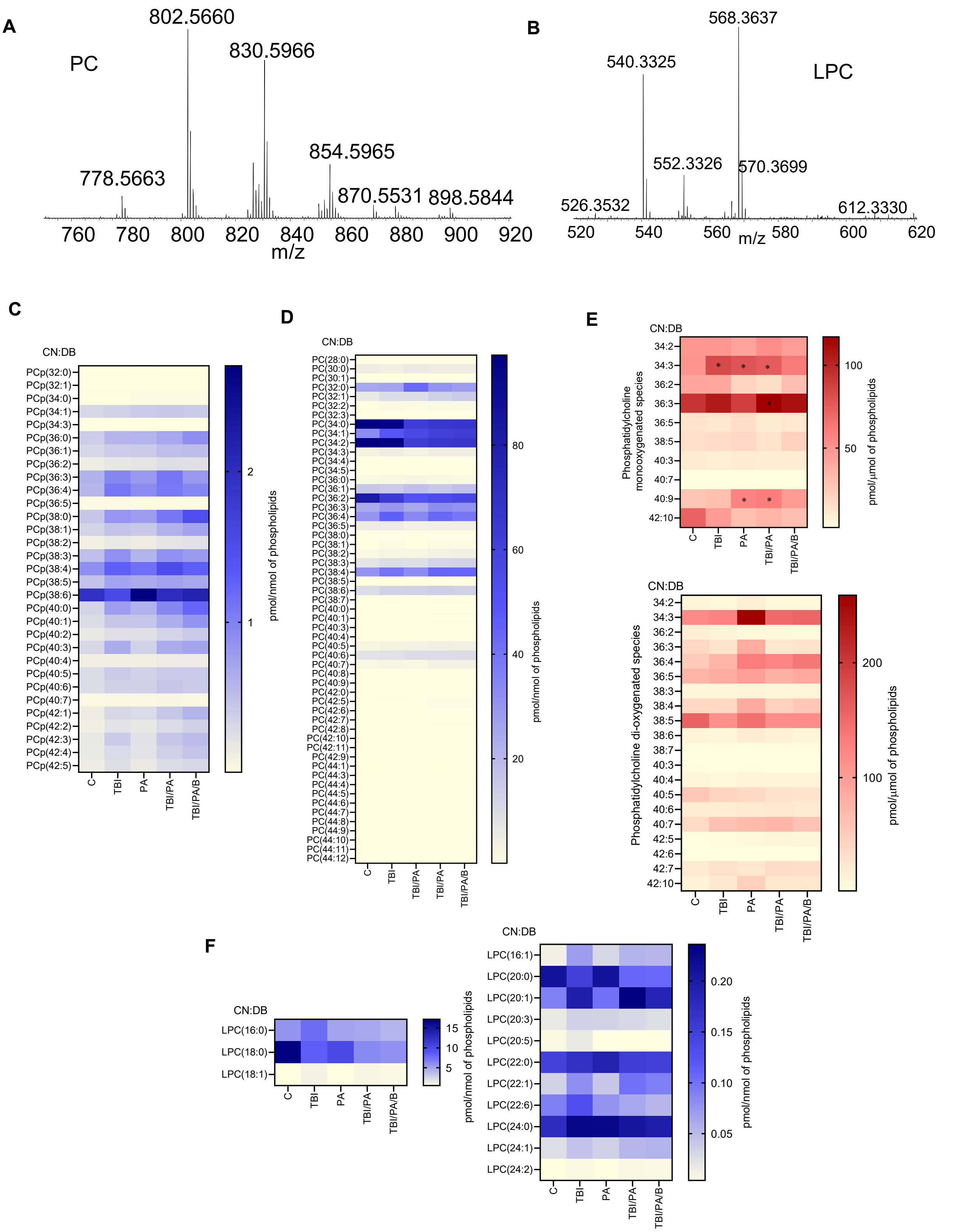

Supplementary Figure 3. Related to Figure 2. Typical full mass spectra of phosphatidylcholine (PC) (**A**) and lyso-phosphatidylcholine (LPC) (**B**) obtained from ileum of control mouse. PC was detected as adduct with formic acid in negative mode. Heat maps showing the content of PC plasmalogen (**C**) , PC di-acyl (**D**), oxygenated PC (**E**) and LPC (**F**) molecular species in ileum of mice. n=5. PC, oxygenated PC and LPC species are labeled as CN:DB where CN is carbon number in sn-1 and sn-2 positions and DB is a double bond number in acyl chains. C - control; TBI - total body irradiation; PAO1 – *P. aeruginosa*; B – baicalein.

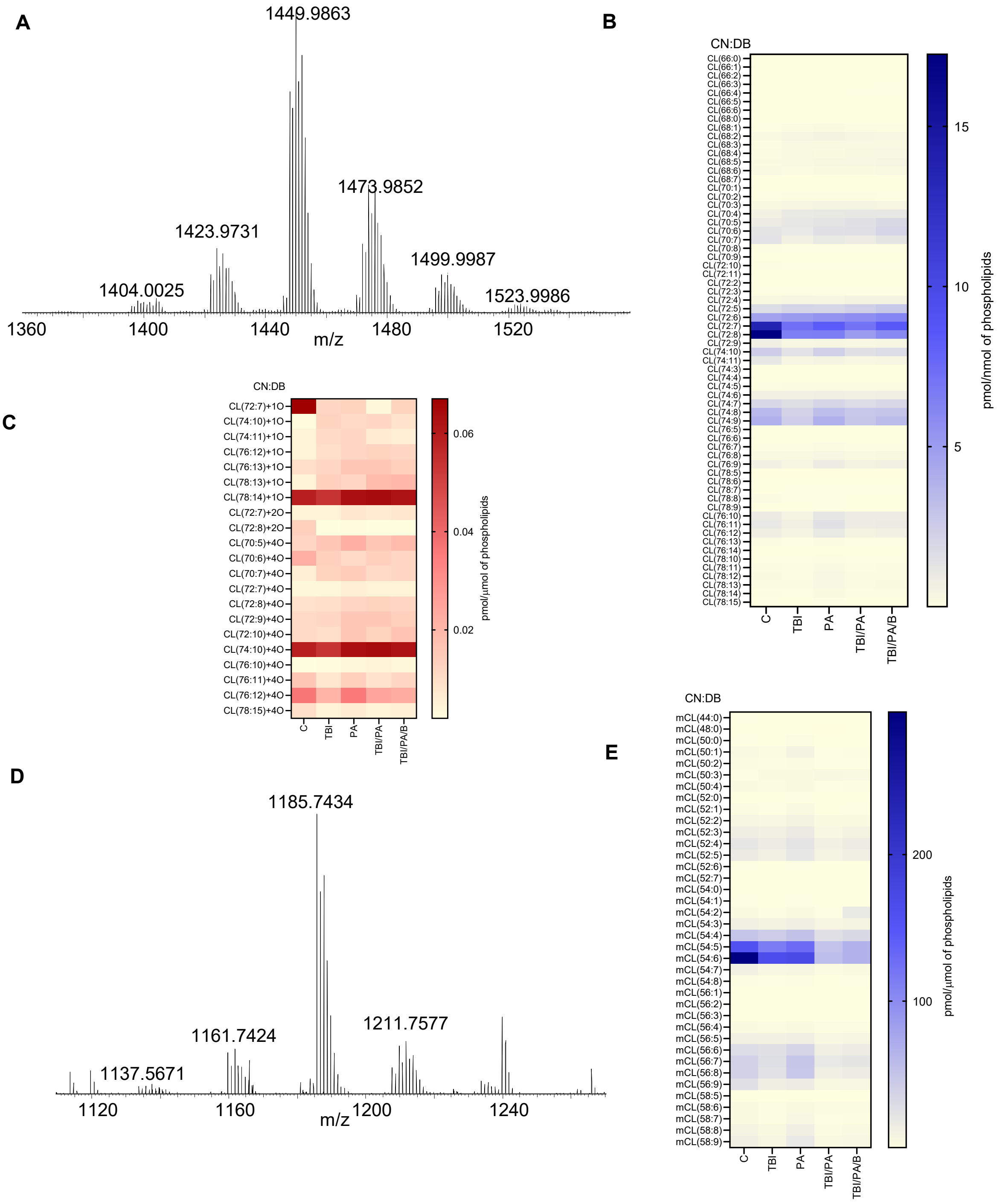

Supplementary Figure 4. Related to Figure 2. Typical full mass spectrum of CL (negative mode) obtained from ileum of control mouse (**A**). Heat maps showing the content of CL (**B**) and oxygenated CL(**C**). Typical full mass spectrum of mono-lyso-CL (mCL) obtained from ileum of control mouse (**D**). Heat map showing the content of mCL (**E**) molecular species in ileum of mice. C - control; TBI - total body irradiaion; PA – *P. aeruginosa*; B – baicalein.

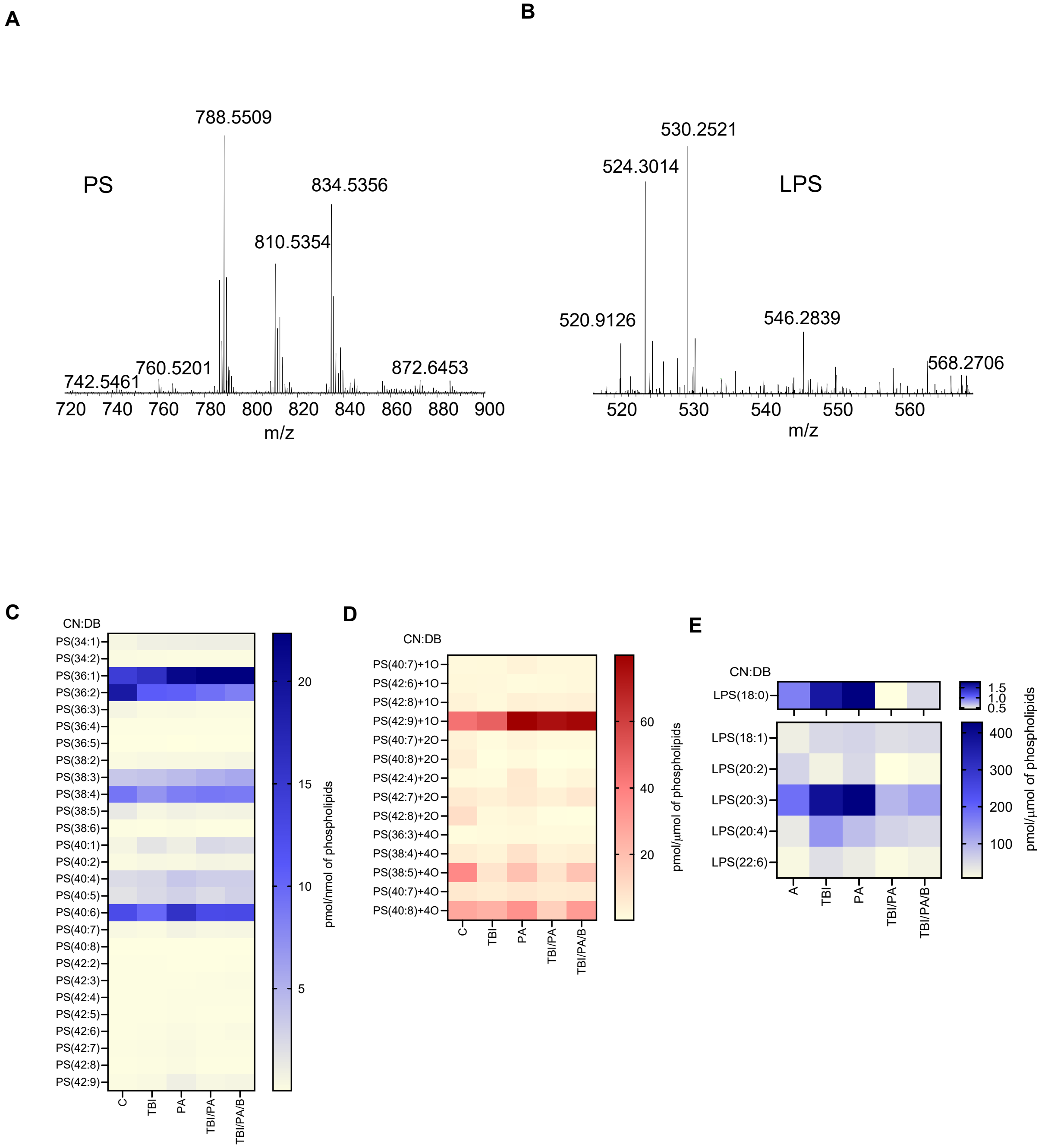

Supplementary Figure 5. Related to Figure 2. Typical full mass spectra of phosphatidylserine (PS) (**A**) and lyso-phosphatidylserine (LPS) (**B**) obtained from ileum of control mouse. Spectra were acquired in negative mode. Heat maps showing the content of PS (**C**), oxygenated PS (**D**) and LPS (**E**) molecular species in ileum of mice. PS, oxygenated PS and LPS species are labeled as CN:DB where CN is carbon number in sn-1 and sn-2 positions and DB is a double bond number in acyl chains. n=5. C - control; TBI - total body irradiation; PAO1 – *P. aeruginosa*; B – baicalein.

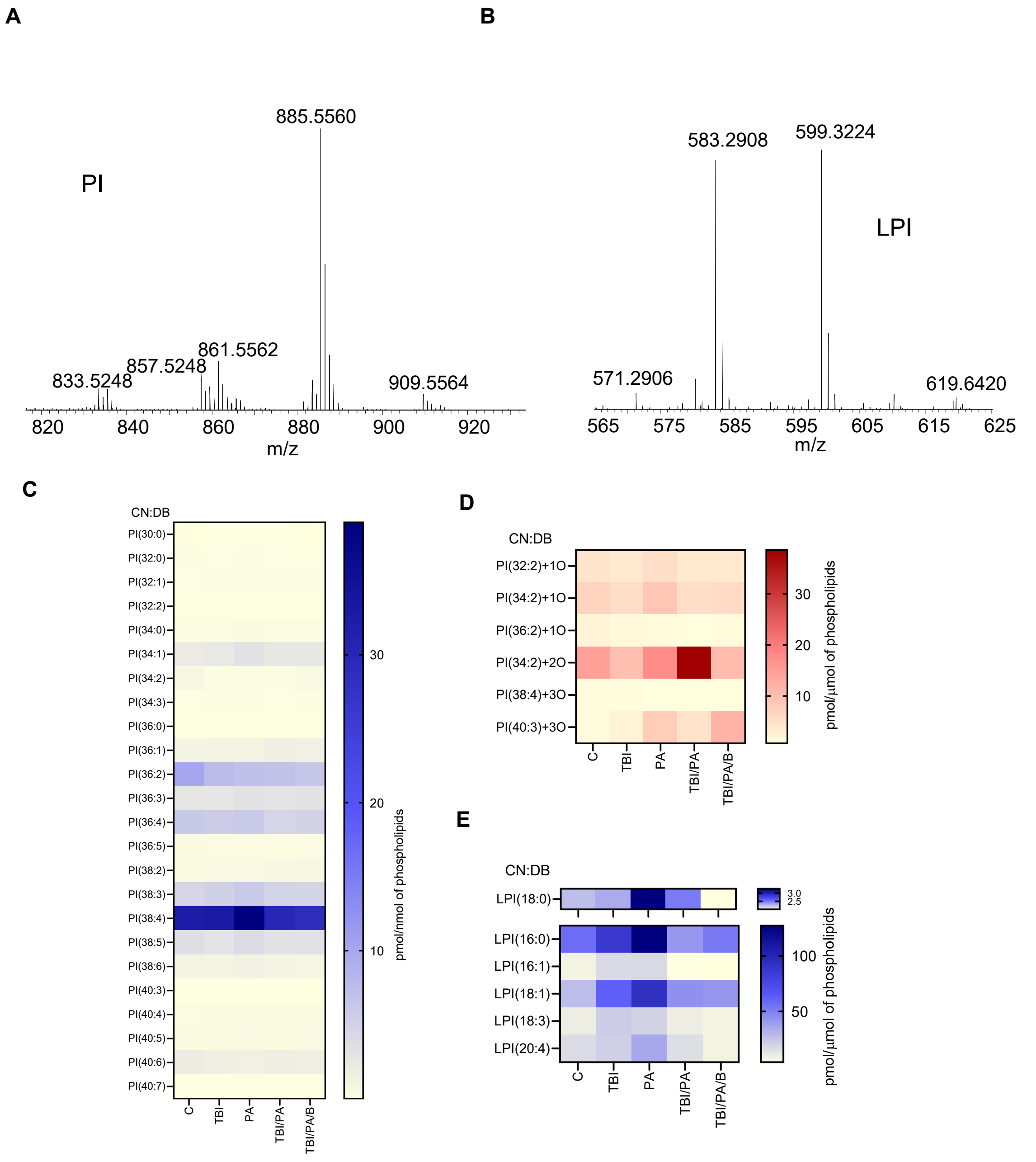

Supplementary Figure 6. Related to Figure 2. Typical full mass spectra of phosphatidylinositol PI (**A**) and lyso-phosphatidylinositol (LPI) (**B**) obtained from ileum of control mouse. Spectra were acquired in negative mode. Heat maps showing the content of PI (**C**), oxygenated PI (**D**) and LPI (**E**) molecular species in ileum of mice. PI, oxygenated PI and LPI species are labeled as CN:DB where CN is carbon number in sn-1 and sn-2 positions and DB is a double bond number in acyl chains. n=5. C - control; TBI - total body irradiation; PAO1 – *P. aeruginosa*; B – baicalein.

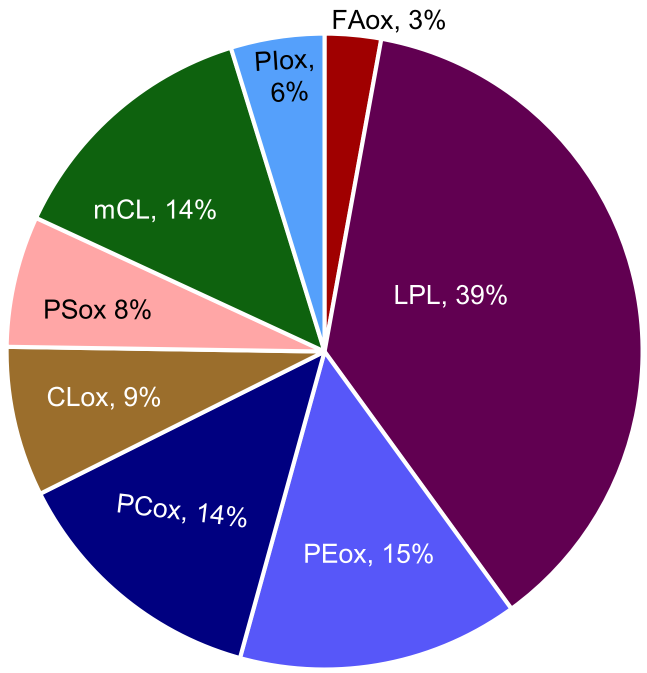

Supplementary Figure 7. Related to Figure 2. Pie-chart showing the contribution of oxygenated lipids in OPLS-DA model. LPL - lysophospholipids; PEox - oxidized phosphatidylethanolamine; PCox – oxidized phosphatidylcholine; CLox – oxidized cardiolipin; PSox – oxidized phosphatidylserine; mCL – mono-lyso-cardiolipin; PI-ox – oxidized phosphatidylinositol; FAox – oxidized free fatty acids. .

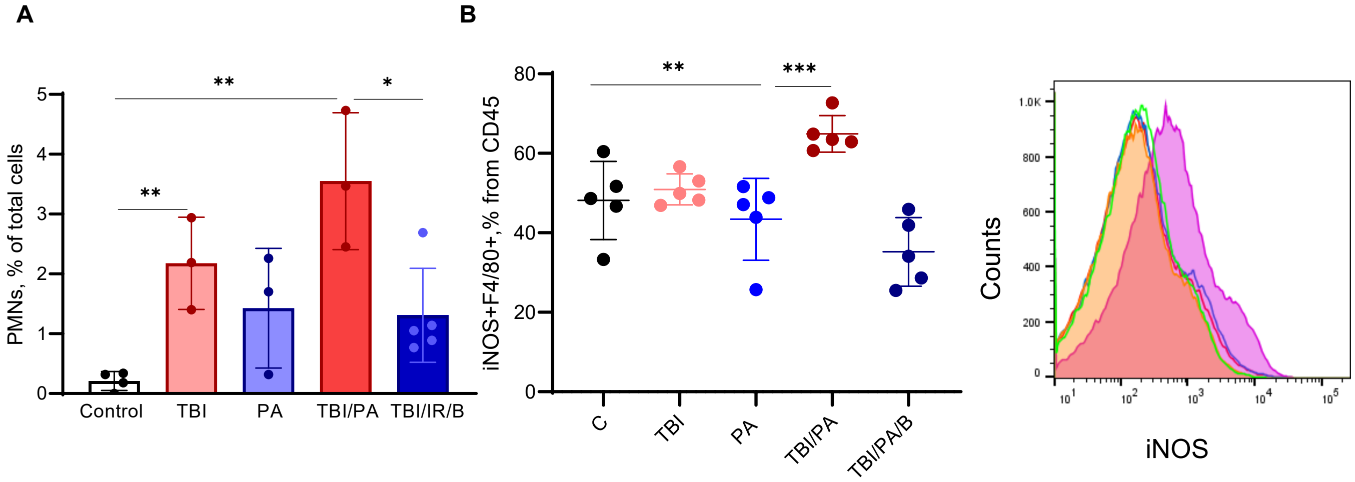

Supplementary Figure 8. Infiltration of immune cells into the ileum on day 4 after TBI. CD45+ cells were collected and then stained with fluorescent-labeled antibodies CD11b, Ly6G (**A**, neutrophils) or iNOS, F4/80 (**B**, macrophages) and analyzed by flow cytometer. Data means ± s.d, n = 3-5 mice/group. *p<0.05, **p< 0.01, ***p< 0.001, 1-way ANOVA Tukey's multiple comparison test.

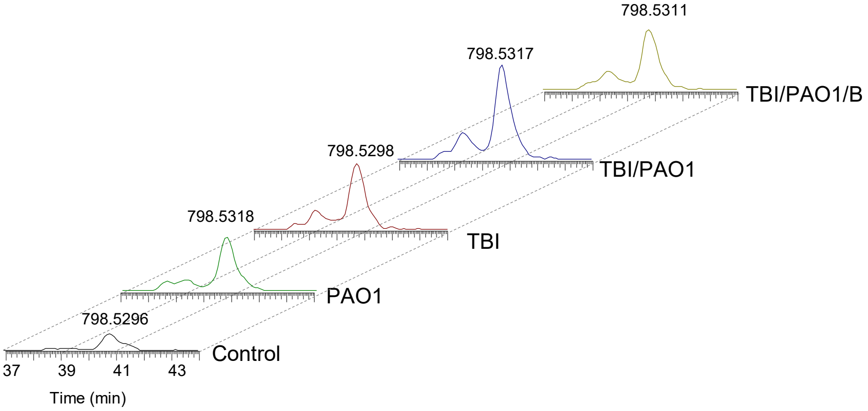

Supplementary Figure 9. Related to Figure 4. Typical base-peak chromatograms of molecular ion with m/z 798.5298 corresponding to 1-SA-2-15-HpETE-PE, a ferroptotic cell death signal, obtained on day 4 from ileum of control mice, mice infected with PAO1, mice exposed to TBI, PAO1 infected mice exposed to TBI and PAO1 infected mice exposed to TBI and treated with baicalein. TBI - total body irradiation; PAO1, *P. aeruginosa*; B, baicalein.

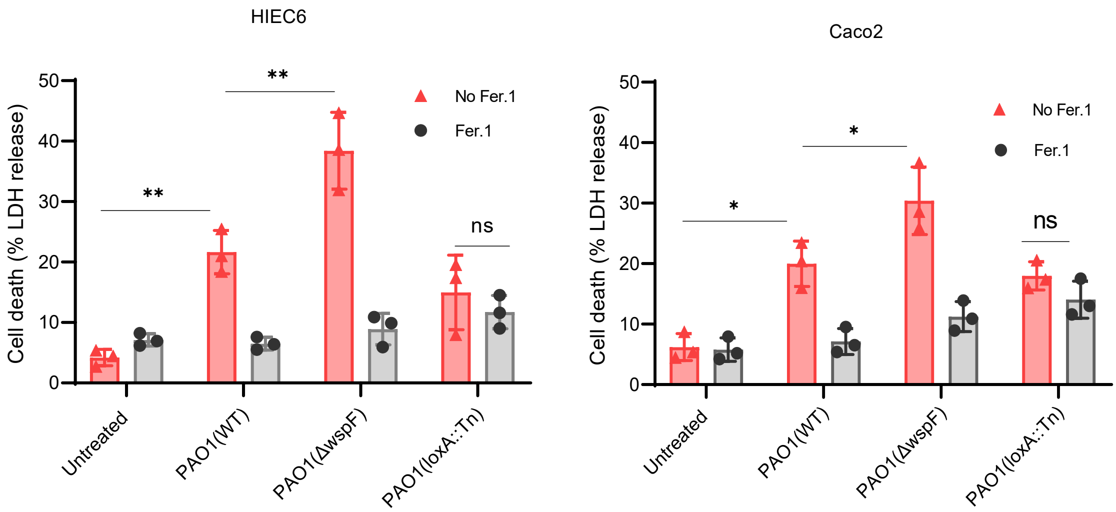

Supplementary Figure 10. PAO1 induced ferroptosis of intestinal epithelial cells is pLoxA dependent. Related to Figure 4**.** PAO1 supernatant was incubated with intestinal epithelial cells. (left panel) HIEC6 cells and (right panel) Caco-2 cells with or without Fer.1 (0.4 μM). Cell death was assessed by LDH release assay after 20 h. Data represents mean ± s.d., $n = 3$. 1-way ANOVA, Tukey's multiple comparison test. $^{**}p < 0.01$, $^{*}p < 0.05$.

Unedited Gel used for Figure 4B.

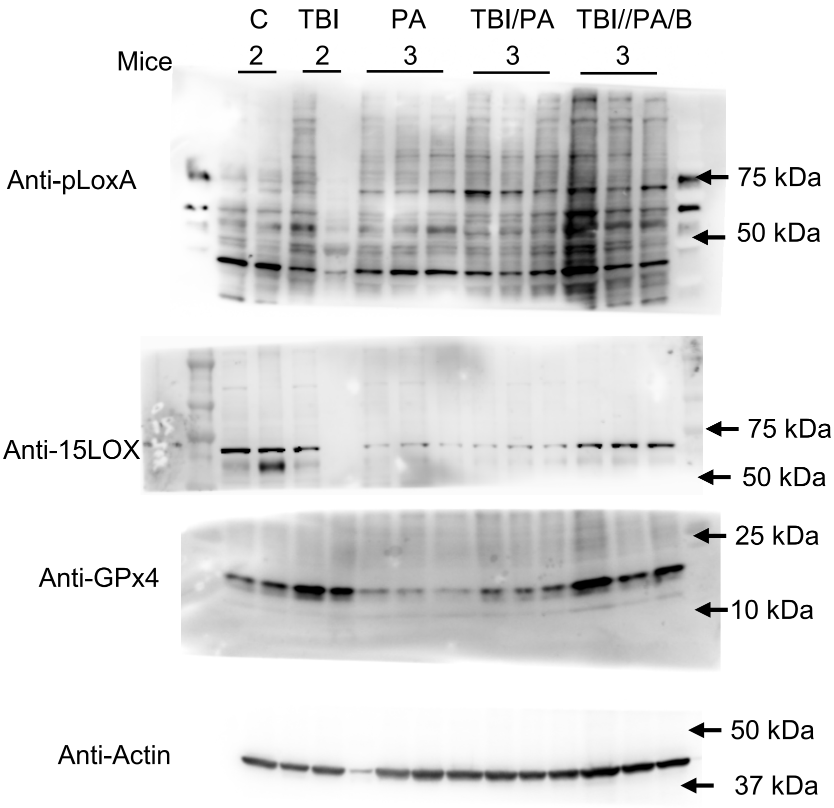

Supplement: Supplemental data [file jciinsight-7-156013-s271.pdf]
